# Transcriptional identification and characterization of differentially expressed genes associated with embryogenesis in radish (*Raphanus sativus* L.)

**DOI:** 10.1038/srep21652

**Published:** 2016-02-23

**Authors:** Lulu Zhai, Liang Xu, Yan Wang, Xianwen Zhu, Haiyang Feng, Chao Li, Xiaobo Luo, Muleke M. Everlyne, Liwang Liu

**Affiliations:** 1 National Key Laboratory of Crop Genetics and Germplasm Enhancement, College of Horticulture, Nanjing Agricultural University, Nanjing 210095, P.R. China; 2 College of Plant Science, Jilin University, Changchun 130062, P.R. China; 3 Department of Plant Sciences, North Dakota State University, Fargo, ND 58108, USA

## Abstract

Embryogenesis is an important component in the life cycle of most plant species. Due to the difficulty in embryo isolation, the global gene expression involved in plant embryogenesis, especially the early events following fertilization are largely unknown in radish. In this study, three cDNA libraries from ovules of radish before and after fertilization were sequenced using the Digital Gene Expression (DGE) tag profiling strategy. A total of 5,777 differentially expressed transcripts were detected based on pairwise comparison in the three libraries (0_DAP, 7_DAP and 15_DAP). Results from Gene Ontology (GO) and pathway enrichment analysis revealed that these differentially expressed genes (DEGs) were implicated in numerous life processes including embryo development and phytohormones biosynthesis. Notably, some genes encoding auxin response factor (*ARF *), Leafy cotyledon1 (*LEC1*) and somatic embryogenesis receptor-like kinase (*SERK *) known to be involved in radish embryogenesis were differentially expressed. The expression patterns of 30 genes including *LEC1-2*, *AGL9*, *LRR*, *PKL* and *ARF8-1* were validated by qRT-PCR. Furthermore, the cooperation between miRNA and mRNA may play a pivotal role in the radish embryogenesis process. This is the first report on identification of DEGs profiles related to radish embryogenesis and seed development. These results could facilitate further dissection of the molecular mechanisms underlying embryogenesis and seed development in radish.

Although plant embryogenesis is usually studied in the context of seed development, there are many alternative routes to embryo initiation, including somatic embryogenesis, microspore embryogenesis and zygotic embryo initiation^1^. These modes of embryogenesis are a crucial developmental period in life cycle of flowering plants^2^,^3^. During embryogenesis, the single-celled zygote, the product of fertilization of female gamete with male gamete, follows a defined pattern of cell division and differentiation to the mature embryo^4^. The developmental events are highly conserved among diverse angiosperms, playing an important role in defining key aspects of seed formation and diversity^5^,^6^. Embryo development consisted of two distinct phases including early morphological events and late maturation^7^. Recent studies demonstrated that early embryogenesis could be considered as a miniature model of plant development based on hallmarks of several key embryonic developmental processes^8^,^9^. However, the molecular events related to plant early embryogenesis are largely unknown compared with other biological processes, which is partially due to poor accessibility and tiny size of embryos in many higher plants^2^.

The morphogenesis phase of embryogenesis is well described in model plants such as *Arabidopsis thaliana*^10^,^11^. In addition, embryogenesis-related genes have been identified in *Arabidopsis*^2^,^3^, *Brassica napus*^12^, *Oryza sativa*^9^, *Gossypium hirsutum*^13^ and *Cucumis sativus*^14^. For example, Tzafrir *et al.* identified 244 candidate *EMB* genes in *Arabidopsis* required for normal embryo development using embryo-defective mutants^15^. Among these EMB genes, the *BABY BOOM* (*BBM*) gene, a transcription factor of AP2/ERF family, was originally isolated as a marker for embryogenic cells in tissue culture^16^. Ectopic expression of *BBM* can induce spontaneous somatic embryos and cotyledon-like structure formation from the vegetative tissues of young seedlings^17^.

Similar to the phenotypes observed in the transgenic lines with overexpression of *BBM*, ectopic or spontaneous embryogenesis phenotypes have been reported in lines with overexpression of the *LEAFY COTYLEDON1* (*LEC1*), *LEC2*, *FUSCA3* (*FUS3*), *ABA INSENSITIVE3* (*ABI3*) and *WUSCHEL* (*WUS*) genes in *Arabidopsis*^12^. Stone *et al.* reported that *LEC1* and *LEC2* are required genetically for the completion of normal seed development and may have complementary but partially redundant functions in embryo formation in *Arabidopsis*^18^, while *ABI3* is involved in embryo maturation. For instance, it could promote transcription of *LEC2*, *FUS3* and *ABI3*, as well as regulated transcription of a member of subfamily *B-3* of the *Apetala2*/*Ethylene Response Factor* (*AP2/ERF*) transcription factors^3^,^19^. Moreover, it was reported that the *Agamous-like15* (*AGL15*) was expressed in embryogenic cultures, which was further integrated into different signaling processes during embryogenesis^20^. Schmidt *et al.* originally identified somatic embryogenesis receptor-like kinases (*SERKs*)^21^, which expressed from the eight-celled stage through to the globular stage, while no transcript was detected in unfertilized flowers.

Radish, *Raphanus sativus* L. (2n = 2x = 18), is a major root vegetable crop of the Brassicaceae family. The thick roots and fresh sprouts are harvested as vegetables. While in North America, radish seeds are produced for oil production. Seeds contain the embryo that develops into an adult plant^1^,^22^. Thus, the embryogenesis is crucial for seed production, quality and yield of seed reproduction in elite seed breeding program for the root crops and the oilseeds in radish^22^. To enhance the selfing seeding rate, it is necessary to understand the molecular processes leading to the early embryonic process in radish. However, little is known about the genetic basis of radish embryogenesis.

The complex physiological and molecular processes during plant embryogenesis result from an intricate program of gene expression^23^. Thus, gene expression study can provide an entry point to analyze the plant embryogenesis processes. With the advent in sequencing technology, an ever increasing number of studies have been conducted to obtain systemic transcript profiles during plant embryogenesis using RNA-seq^24^. Nevertheless, there is no published study on identification of differentially expressed transcripts during radish embryogenesis. In this study, a comprehensive radish transcriptomes and microRNA (miRNA) expression profiles were obtained during embryogenesis and the early development of seed development using RNA-seq. Genes associated with transcriptional regulation, signaling pathways and metabolic pathways were over-represented among differentially expressed genes (DEGs) in radish embryogenesis and seed development. Based on an integrative analysis of embryogenesis related miRNAs and DEGs, a schematic model of radish embryogenesis was proposed.

## Results

### Properties of the sequenced cDNA libraries

**Figure 1:**
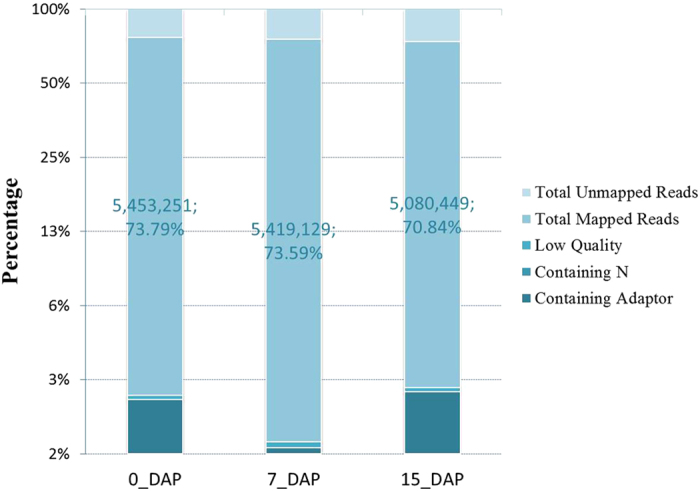
The quality assessment of sequencing data from 0_DAP, 7_DAP and 15_DAP libraries. The large portion per bar showed total number and percentage of reads mapped to the reference sequences.

To identify genes involved in radish embryogenesis, three cDNA libraries were constructed from tissues of ovules at 0, 4–7 and 10–15 day after pollination (DAP). A total of 7,191,808, 7,235,080 and 6,963,130 reads were generated from these three libraries (0_DAP, 7_DAP and 15_DAP, respectively) (Table 1), from which 73.79%, 73.59% and 70.84% reads could be successfully mapped to the reference genome (Fig. 1). The perfect match reads were 3,874,571 (0_DAP), 3,871,479 (7_DAP) and 3,557,884 (15_DAP). To evaluate the quality of the RNA-seq data, the distribution of unigene coverage in each sample was analyzed, which was the number of clean reads that aligned to the reference genes. As showed in Supplementary Fig. S1, the distributions of the transcripts obtained in the three libraries (0_DAP, 7_DAP and 15_DAP) were uniform, and most of which covered more than 50% reference genes (0_DAP, 57% of all genes; 7_DAP, 52% of all genes; 15_DAP, 51% of all genes; Supplementary Table S1), implying that the transcript sequences detected by RNA-seq technology are reliable.

### Identification of differentially expressed transcripts

**Figure 2:**
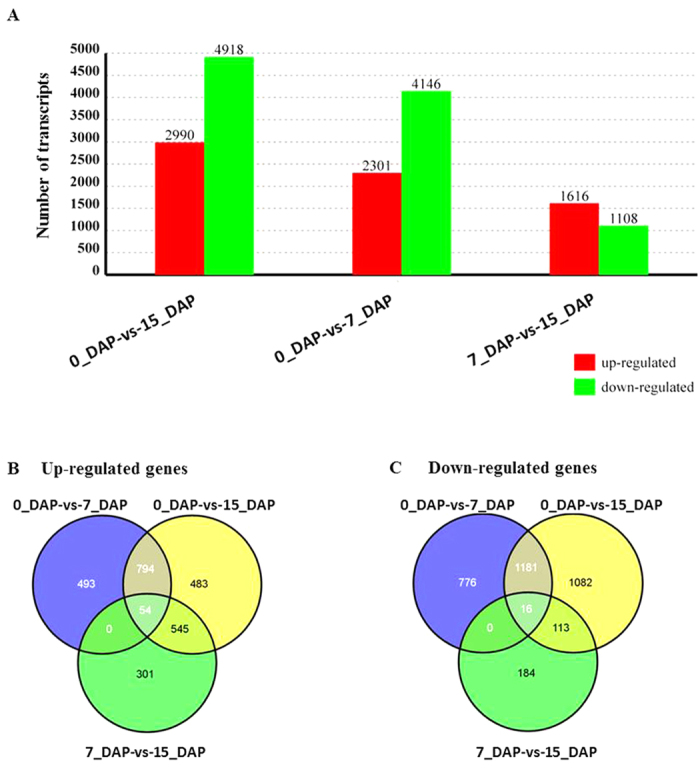
Differentially expressed transcripts during radish embryogenesis (0_DAP, 7_DAP and 15_DAP). (**A**) Number of differentially expressed transcripts in three libraries (0_DAP, 7_DAP and 15_DAP) based on pairwise comparison. Numbers of up- and down-regulated genes were summarized; (**B**, **C**) Number of commonly differentially expressed transcripts among the three libraries (0_DAP, 7_DAP and 15_DAP). (**B**, **C**) showed the number distribution of up- and down-regulated transcripts based on pairwise comparison, respectively.

By comparing our three Illumina libraries, a great number of differentially expressed transcripts were identified that were likely involved in radish embryogenesis. The expression levels of genes in three libraries were analyzed by determining the number of reads per kb per million (RPKM) of clean tags. Using the false discovery rate (FDR) ≤ 0.001 and |log_2_Ratio| ≥ 1 as the threshold values, a total of 5,777 transcripts which accounted for approximately 11.17% of the total transcripts present in three libraries were differentially expressed during radish embryogenesis based on pairwise comparison between the three libraries (Fig. 2A and Supplementary Fig. S2). Between library 0_DAP and 15_DAP, 2,990 and 4,918 transcripts were up- and down- regulated, respectively. A total of 2,301 transcripts were up-regulated and 4,146 transcripts were down-regulated between 0_DAP and 7_DAP library. The complete list of the DEGs during embryogenesis in radish was shown in Supplementary Table S2. Commonly up- and down-regulated transcripts were identified among embryogenesis and seed development stages to determine the degree of overlap (Fig. 2B, C). Maximum number of commonly up- (848) and down- (1197) regulated transcripts were observed between 0_DAP-vs-7_DAP and between 0_DAP-vs-15_DAP comparisons, respectively (Fig. 2B, C).

### Cluster analysis of differentially expressed transcripts

**Figure 3:**
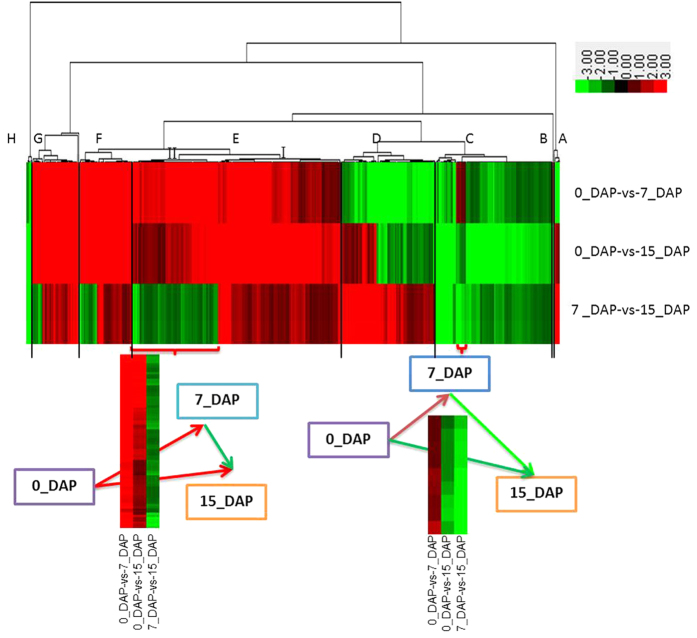
Cluster diagram of DEGs based on pairwise comparison among the three libraries (0_DAP, 7_DAP and 15_DAP) in radish. Expression levels for DEGs for the three groups of pairwise comparisons were hierarchically clustered and shown in a heat-map. Level of expression was represented by color scale from green (down-regulated expression) to red (up-regulated expression), as indicated by a scale bar in the upper right corner. The dendrogram of distances were also shown for genes, names of which were presented in Supplementary Table S3. The two small heat-maps placed lower showed the enlarged view of the two sections in red braces.

To explore patterns of co-regulation of the DEGs during embryogenesis and seed development in radish, expression profiles of these genes were clustered using the hierarchical clustering algorithm and visualized in a heat map. The expression patterns were separated into eight major clusters (A–H) (Fig. 3). Transcripts present in each of the three libraries within each cluster were shown in Supplementary Table S3. Among these major clusters, the up-regulated transcripts were enriched in cluster A, D, E, F and G, while the down-regulated transcripts were enriched in cluster B and C. All the six transcripts in cluster A, 85 out of 87 in cluster D, 112 out of 191 in cluster E, 32 out of 49 in cluster F were up-regulated at the late stage (15_DAP) of radish embryogenesis. Moreover, among these transcripts, the differentially expressed genes in cluster E (II) including WRKY22, zinc finger homeodomain protein 1, efflux carrier of polar auxin transport, lipoxygenase 2 and beta glucosidase 18, were expressed at a higher level during early (7_DAP) embryogenesis, implying that these genes may play a key role in initial embryo development and morphogenesis phase in radish. Only one transcript in cluster B, and nine out of 107 transcripts in cluster C were up-regulated in 0_DAP-vs-7_DAP, whereas all the genes in cluster H were down-regulated in any pairwise comparison of libraries (Fig. 3).

### GO classification and enrichment analysis of the DEGs

**Figure 4:**
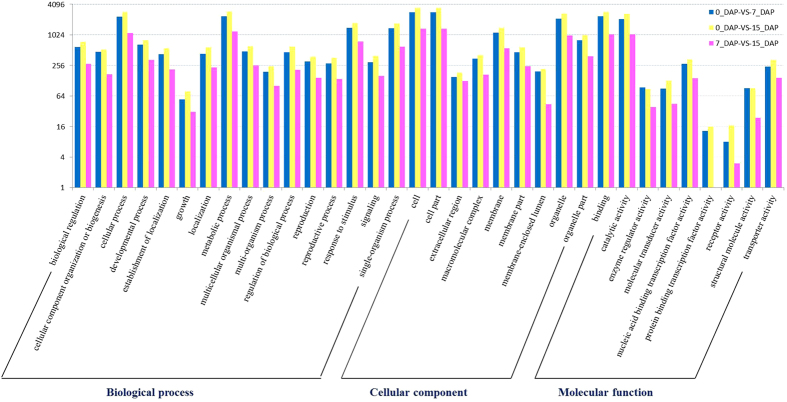
Histogram of level 2 Gene ontology classification of DEGs during embryogenesis in radish. Results are summarized for three main GO categories: biological process (P), molecular function (F), and cellular component (C). The x-axis and y-axis indicate the names and the number of each GO term, respectively.

To get better understanding of the biological functions of these transcripts during radish embryogenesis, DEGs were annotated with Gene Ontology (GO) categories based on GO assignments of top matches in the NCBI non-redundant (nr) database. Based on sequence similarities, 4,513 (0_DAP-vs-7_DAP), 5,576 (0_DAP-vs-15_DAP) and 2,091 (7_DAP-vs-15_DAP) DEGs were categorized into 34 GO terms, respectively (Fig. 4). Among GO term, the ‘metabolic process’, ‘cell’, ‘cell part’, ‘organelle’, ‘binding’ and ‘catalytic activity’ terms were dominant among DEGs. Only a few of the DEGs between 0_DAP and 7_DAP as well as 0_DAP and 15_DAP were classified in the categories ‘protein binding transcription factor activity’, whereas no DEG between 7_DAP and 15_DAP was annotated.

According to GO functional enrichment analysis at early stage of embryogenesis, a total of 6 (cellular component), 16 (molecular function) and 54 (biological process) terms were significantly enriched (Bonferroni-corrected *P*-value ≤ 0.05; Supplementary Table S4). The enrichment of the genes participated in various metabolic and reproductive processes including cell wall (GO:0005618), post-embryonic morphogenesis (GO:0009886), embryonic axis specification (GO:0000578), embryo development (GO:0009790), regulation of post-embryonic development (GO:0009791), embryonic pattern specification (GO:0009880), embryonic morphogenesis (GO:0048598) and floral organ development (GO:0048437).

### Functional classification using the KEGG database

To further identify the functions of DEGs during embryogenesis and seed development, functional classification and pathway assignment of DEGs were performed using the KEGG database with a hypergeometric test and Bonferroni Correction^25^. In total, 127 KEGG pathways had at least one DEG. As shown in Table 2, the annotated DEGs were predominantly enriched in 20 KEGG pathways (Supplementary Table S5), particularly plant hormone signal transduction [ko04075], starch and sucrose metabolism [ko00500], brassinosteroid biosynthesis [ko00905] and phenylpropanoid biosynthesis [ko00940], indicating that these were the active pathways during radish embryogenesis and seed development. Plant hormone signal transduction contains several biosynthesis and metabolic patterns, including Tryptophan metabolism (ko00380, seven DEGs), Zeatin biosynthesis (ko00908, four DEGs), Brassinosteroid biosynthesis (ko00905, six DEGs), Diterpenoid biosynthesis (ko00904, two DEGs), Carotenoid biosynthesis (ko00906, four DEGs), Cysteine and methionine metabolism (ko00270, three DEGs), α-Linolenic acid metabolism (ko00592, three DEGs) and Phenylalanine metabolism (ko00360, two DEGs) (Supplementary Fig. S3). Some DEGs involved in these plant hormone signal transduction pathways were shown in Table 3.

### Experimental validation of DEGs through qRT-PCR

**Figure 5:**
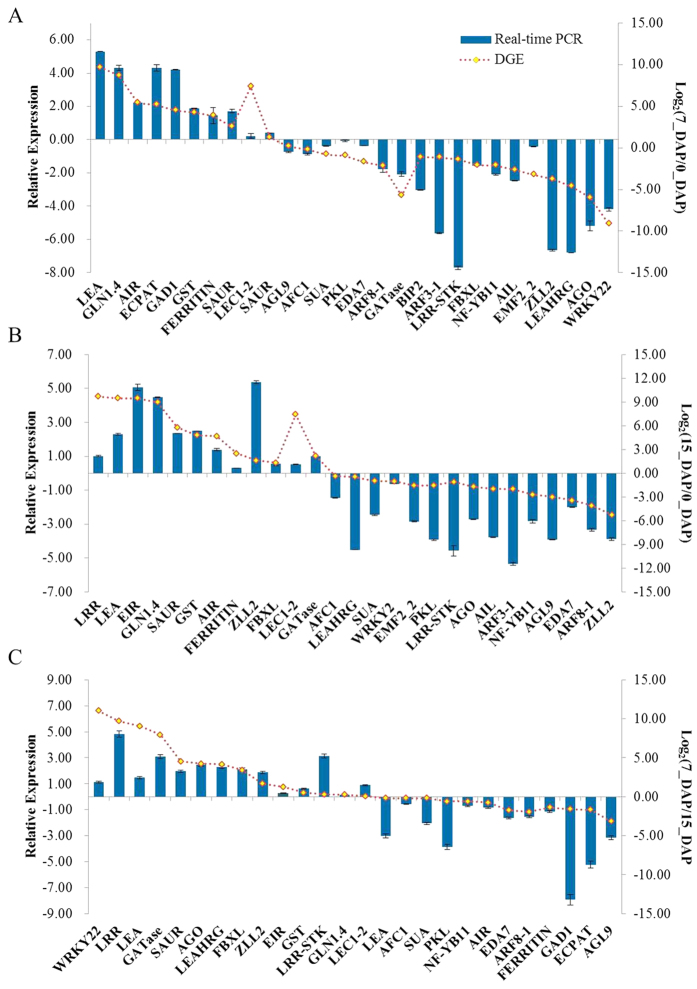
RT-qPCR validation of some DGEs during radish embryogenesis. The relative expression of DGEs between 0_DAP and 7_DAP libraries (**A**), 0_DAP and 15_DAP libraries (**B**) and 7_DAP and 15_DAP libraries (**C**) were analyzed by the 2^−ΔΔ*C*T^ method.

To evaluate the validity of Illumina sequencing analysis and further assess the patterns of DEGs, 28, 27 and 26 genes were selected from the three pairwise comparisons (0_DAP-vs-7_DAP, 0_DAP-vs-15_DAP, 7_DAP-vs-15_DAP) for qRT-PCR analysis, respectively (Fig. 5). For all of the selected genes, the expression patterns determined in real-time RT-PCR were in agreement with those based on RNA-seq. Nonetheless, there were discrepancies with respect to degrees of differential expression between the two methods. This might be attributed to the differences in the algorithms used to determine expression levels. The high-throughput sequencing technique generated absolute rather than relative expression measurements. Furthermore, to visualize the correlations intuitively, real-time RT-PCR was performed on 17 DEGs in three comparative groups (0_DAP-vs-7_DAP, 0_DAP-vs-15_DAP and 7_DAP-vs-15_DAP), which showed different expression profiles during early embryonic process, with high or low expression levels at one or more time points. The Pearson correlation coefficient was calculated by SPSS to assess the correlation between different platforms (Supplementary Fig. S4). Overall, we observed a good concordance in the expression patterns of DEGs obtained by both the RNA-seq and qRT-PCR method as indicated by the overall correlation coefficient (0.84), implying the reliability of the transcriptomic profiling data.

### DEGs involved in embryo induction, formation and maturation stages

To understand molecular mechanism of embryogenic competence acquisition embryogenesis and seed development, the DEGs with a higher degree of differential expression (|log_2_Ratio| ≥ 2) were further characterized (Supplementary Table S6). Several differentially expressed transcription factors, transporters, hormone response/transduction genes and transcripts encoding embryo defective protein etc. were identified, including *LEA* (*late embryogenesis abundant protein*), *SERK* (*somatic embryogenesis receptor-like kinase*), *EIR* (auxin efflux carrier family proteins), *ARF*s (*auxin response factor*), *ANT* (*aintegumenta*), *GST* (*glutathione S-transferase*), *WRKY*s (WRKY transcription factors), *FERRITIN*, *AG/ZLL2* (argonaute/Zwille-like proteins) and *AGL*s (Agamous-like MADS-box proteins).

**Figure 6:**
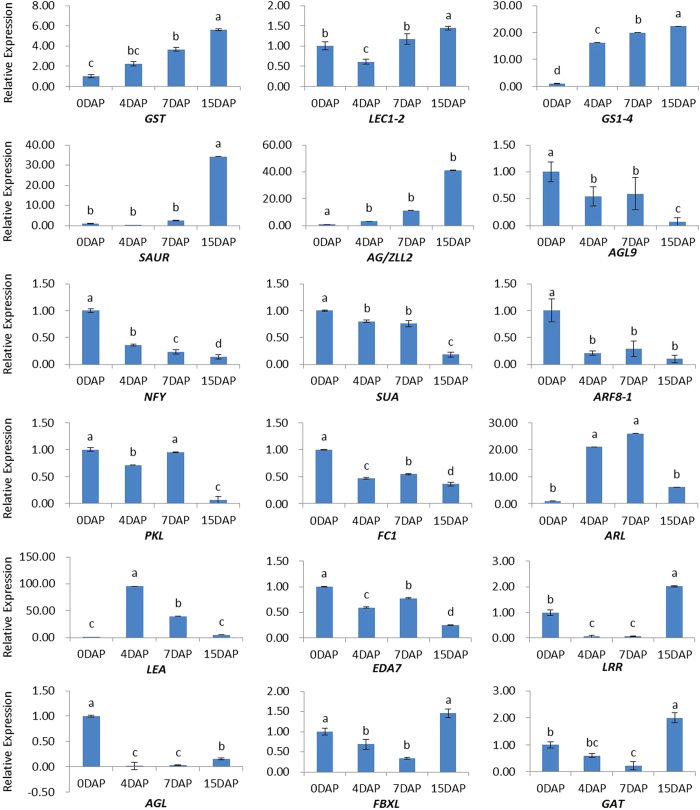
Relative expression levels of DEGs in four developmental stages of radish embryogenesis. The normalized expression levels at 0 DAP were arbitrarily set to 1. Different letters indicate significant differences at *P* < 0.05 according to Duncan’s multiple range tests. Each bar shows the mean ± SE of triplicate assays.

To further investigate and verify the expression variations of the DEGs, transcriptional qRT-PCR analysis of 18 selected genes including *AGL9*, *ARF8-1*, *LEC1-2*, *LEA* protein, *NF-Y* (nuclear factor Y) and *GST*, were performed for a more detailed analysis of ovule gene expression after pollination in different days (0 DAP, 4 DAP, 7 DAP and 15 DAP). Among these genes associated with embryo induction and formation, they can be classified into four groups. The first group including *GST*, *LEC1-2* transcription factor, *GS1-4* (glutamine synthetase cytosolic isozyme 1–4), *SAUR* (small auxin up RNA protein), *AG/ZLL2*, showed an up-regulated expression during the embryo induction and formation stages (Fig. 6). DEGs in the second group were mostly transcription factors where some were reported previously as important regulators of plant developmental process, including *AGL9*, *NF-Y*, *SUA* (suppressor of *ABI3-5*), *ARF8-1*, *PKL* (PICKLE) and *FC1* (*fus3*-complementing gene 1). In addition, the expression of *ARL* (auxin-responsive-like protein) and *LEA* reached a peak at 4–7 DAP ovule, suggesting that these genes may play an important role in morphogenesis and maturation of embryos. Some other genes in group 4 including *EDA7* (embryo sac development arrest 7 protein), *LRR* (leucine-rich repeat-containing protein), *AGL*, *FBXL* (F-box/LRR-repeat protein) and *GAT* (glutamine amidotransferase) were found to be down-regulated during early embryogenesis and up-regulated slightly in the late stage. These observations showed that some transcription factors and genes, such as *LEC1-2*, *ARFs* and *AGL*s, were responsive to phytohormone, which could be involved in normal morphogenesis phase of zygotic embryogenesis in radish.

### Integrative analysis of DEGs and miRNAs during embryogenesis

**Figure 7:**
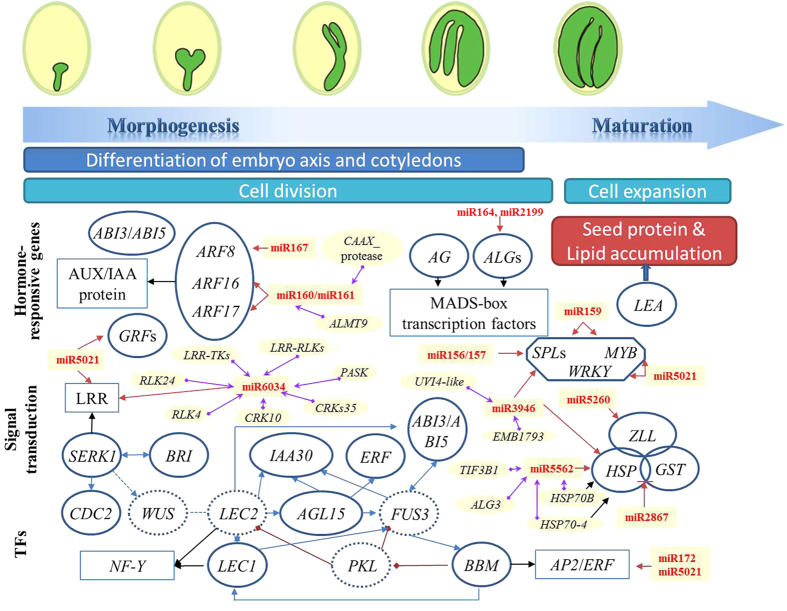
A proposed model of genetic and molecular interactions in the regulatory network during embryogenesis in radish. Embryo development consists of two phases, morphogenesis and maturation. Arrows in blue and black indicate regulation and affiliation, respectively, whereas red lines between genes indicate transcriptional repression. The circle with solid and dotted line represented genes identified or not in radish and the corresponding miRNAs are shown in red. Genes in yellow boxes were connected with miRNAs by purple arrows using Cytoscape, which were visualized as miRNA-mRNA interactions.

Previous studies have extensively showed that many miRNA-mRNA pairs were involved in embryogenesis, such as miR160/167-*ARF*s, miR156/157-*SPL*s and miR172-*AP2*^26^,^27^,^28^. Based on the previously identified radish embryogenesis-related miRNAs^29^, and the DGE data in this study, we found that the differential expression of several genes likely involved in developmental and physiological events during radish development were modulated, to some extent, by the activity of miRNAs (Fig. 7). Our data sets revealed that 57 candidate genes were involved in regulation of phytohormones (Supplementary Table S7). These genes including *ARF*s, *GH3*, *IAAs*, *ARP* (auxin-repressed protein), *EIR*, *ERF* (ethylene-responsive transcription factor) and *SAUR* were modulated during different stages of embryogenesis, and their expression profiles were correlated with several miRNAs such as miR160/161 and miR167 (Fig. 7), indicating their potential key roles and regulatory relationships during embryogenesis and seed development in radish. Moreover, one *BBM* gene (targeted by miR172 and miR5021), 32 *LRR* genes (targeted by miR5021 and miR6034), 12 *LEA* genes and 3 *AGLs* genes (targeted by miR164 and miR2199) were significantly differentially expressed at different development stages of radish embryo (Fig. 7 and Supplementary Table S7). During embryogenesis, a series of developmental transition steps were orchestrated by transcription factors including those in the *WRKY* gene family^30^. A total of 28 *WRKY* gene transcripts representing 17 family members were identified (Supplementary Table S7).

To gain in-depth insight into the potential relationship between DEGs and embryogenesis-related miRNAs^29^, a co-expression analysis was performed based on the mRNA and miRNA data to identify DEGs that were likely co-regulated with differentially expressed miRNAs. Among them, 75 pairs of miRNA/mRNA were found as co-expressed (Supplementary Table S8) and a partial regulatory network was visualized in Fig. 7. Further analysis showed that 33 pairs containing 19 known miRNAs and 28 unique transcripts showed a negative correlation in expression trends during early embryogenesis, providing valuable information for the regulation of miRNA in the regulatory network of radish embryogenesis.

## Discussion

Embryogenesis represents an elaborate and complex phase in plant life cycles, which condenses the fundamental processes underlying plant development into a short sequence of predictable steps^5^,^8^. Previous studies have identified several genes involved in regulating plant embryogenesis mainly by mRNA differential display (mRNADD)^31^ or hybridization-based approaches^32^. In contrast to the above-mentioned approaches, the RNA-seq technology has clear advantages for transcriptome profiling, which can generate millions of sequence reads with high reproducibility, and create a comprehensive view of the participation of several multi-gene families in plant embryogenesis^33^,^34^. However, no study on comprehensive identification of the DEGs during embryogenesis has yet been conducted in radish. This study was undertaken with the goal to investigate the genes regulating radish embryogenesis and seed development. By pairwise comparisons of the data between three libraries (0_DAP, 7_DAP and 15_DAP), differential transcript abundance and regulation were demonstrated. Genes associated with secondary metabolism, signal transduction, hormone response/transduction and development were identified, and their expression profiles were characterized in detail.

### Transcription regulation underlying embryogenesis in radish

Large-scale transcription analyses of embryogenesis had been demonstrated in several species^12^,^35^. Because multiple transcription factors (TFs) were differentially expressed durng embryogenesis and a number of them have known roles during embryogenesis in other plant models, our study contributes to a better understanding of embryogenesis process^10^,^36^. In this study, at least two groups of TFs (*AP2/ERF* Domain Protein and *MADS*-Domain Protein *AGAMOUS-LIKE*) were present with complex expression profiles. Several members in the *AP2/ERF* family had also been reported to be involved in microspore-, somatic- and zygotic embryogenesis^32^. In this study, the radish orthologs of *APETALA2* (*AP2*, negatively controlling cell proliferation during seed development), *AINTEGUMENTA* (*ANT*, expressing in the primordia of cotyledons) and *BABY BOOM* (*BBM*, promoting cell proliferation and morphogenesis during embryogenesis) were differentially expressed during radish embryogenesis and seed development, consistent with their actions in *Arabidopsis thaliana*^17^, *Brassica napus*^16^, *Capsicum annuum*^37^ and *Elaeisguineensis*^38^. These findings suggested that these TFs potentially participate in the specific process embryogenesis and early seedling development in radish^9^.

The *AINTEGUMENTA-LIKE* (*AIL*) transcription factor genes including *BBM*, *ANT* and *PLETHORA* (*PLT*) genes in the *AP2*/*ERF* family and were expressed in young tissues. These *ANT* genes play key roles in many developmental processes, e.g. embryogenesis, meristem maintenance, organ positioning and growth^39^,^40^. Previous studies showed that *ANT* had a central role in ovule development through the activation of *HD-ZIP III* genes that promote apical fate during early embryogenesis^39^, in accordance with the results obtained in this study. Currently, the *BBM* gene had been regarded as a transcriptional activator or repressor in a wide range of developmental pathways, such as plant embryogenesis, and its targets support a role for AIL proteins in the regulation of cell proliferation and differentiation^39^.

Although some regulators can directly influence embryo identity in somatic tissue, other factors act in a more indirect manner. The latter might involve an increase in the capacity to induce somatic embryos in response to other triggers rather than in inducing embryogenesis directly^1^. The *AGL* genes, belonging to MADS-domain protein family, were detected during radish embryogenesis, which were also reported to express in young developing embryos, promote somatic embryogenesis and enhance production of secondary embryonic tissue in soybean^41^, rice^33^ and Arabidopsis^20^. In the present study, four *AGL* genes were down-regulated, suggesting that they preferentially accumulated during the early stages of radish embryo development and played an important role in the beginning of embryogenesis^42^.

In addition to the *AP2s* and *AGLs*, the *LEC2* and *WUS* homeodomain protein genes were identified but not differentially expressed under the threshold value in our data. The *LEC1* (At-NF-YB9), a member of *NF-Y* family homologous with *LEC1-LIKE* (*L1L*; At-NF-YB6), was also identified in this study. *LEC1* was a general integrator of light and hormone signaling during embryogenesis^43^ and its action included processes beyond the embryo, e.g. etiolation responses in young seedlings^44^. In the present study, the expression levels of *LEC1-2* analyzed by qRT-PCR were low at 4 DAP and gradually increased in later stages. *PKL*, a putative chromatin remodeling factor, showed an opposite expression trend, leading to the hypothesis that *PKL* repressed *LEC1-2* TF during radish embryogenesis. Consistent with *LEC* overexpression phenotypes, *pkl* mutants accumulated storage macromolecules and give rise to embryogenic calli in culture^45^.

### Crucial roles of hormone in embryogenesis and seed development

In dicotyledonous plant, the embryogenesis begins with fertilization of egg and subsequently the zygote undergoes cell elongations and divisions^4^. Plant growth regulators (PGRs), *ARF*s, Aux/IAAs, *SUA* and *ARL* were involved in plant embryogenesis^4^,^36^. In *Arabidopsis*, Xiang *et al.* demonstrated that the auxin signaling events associated with gene activity were more prevalent in early developmental stages when the post-fertilization sporophytic program is initiated^2^. In this study, genes related to auxin biosynthetic and metabolic processes, polar auxin transport, homeostasis and auxin-mediated signaling, were highly expressed during radish embryogenesis and seed development, which was consistent with the observations in rice^46^, cotton^36^ and Arabidopsis^30^,^34^. Auxin is considered to be a critical PGR in cell division and differentiation, and its level surges occurred during embryogenesis with consequent accumulation of numerous mRNAs^9^,^36^. Some highly expressed auxin-related genes, including auxin-responsive family protein (*ARL*), *EIR*, *GH3*s, *PIN*s and small auxin-up RNAs (*SAUR*s) were differentially expressed, suggesting that they might play important roles in development process of radish embryogenesis^9^.

Moreover, the brassinosteroids (*BR*s) are an important class of signaling hormones involved in plant growth and development^47^. In this study, the plant hormone signal transduction “super-pathway” was amongst the most predominant one. This super-pathway includes a total of eight pathways (Supplementary Fig. S3). Among them, *BR* biosynthesis pathway has been widely reported to interact with other phytohormones influencing plant development^48^,^49^. *BR*s are a class of polyhydroxylated steroidphyto hormones essential for plant growth and development, including cell division^47^, cell elongation^50^, root development^48^, flowering time^51^ and embryogenesis^52^. The *BR*s could regulate gene expression through a receptor kinase-mediated signal transduction pathway^53^. In particular, the main BR-perceiving receptor, BR-insensitive1 (*BRI1*) and *BAK1*/*SERK3* are co-receptors for brassinosteroids, and this *SERKs* of the leucine-rich repeat receptor-like kinase subfamily II (*LRR-RLK II*) that are associated with the process of somatic embryogenesis^9^. *BRI1*, was also important for cell fate specification in the stem vasculature and seedling root epidermis^54^,^55^. Further studies were required to unravel the functional effect of brassinosteroids in regulating radish embryogenesis.

### Signal transduction pathway in embryogenesis

The process of embryogenesis involves a series of dramatic transitions due to differential gene expression as well as various signal transduction pathways for activating or repressing gene sets^9^,^12^. A number of studies had reported that the Receptor-Like Kinases (RLKs) and Receptor-Like Cytoplasmic Kinases (RLCKs) are involved in many different signaling processes such as hormone signaling, growth and development and have important functions during embryonic intercellular signaling event^55^,^56^, among which, the *SERK* gene, a member of LRR-RLKs, was one of the most important genes regulating a successive downstream signal transducers in the signal transduction pathway (Fig. 7). Some *SERK* genes were recognized as markers for cell competent to form somatic embryos in culture, but specific functions for the *SERK*s in zygotic embryogenesis were elusive^55^. In the present study, we found a great number of *SERK*s [EC: 2.7.10.1, 2.7.11.1] as a leucine-rich repeat (LRR) transmembrane protein kinase in the KEGG pathway (Supplementary Table S6), which may play a crucial role in triggering embryogenesis in plants^3^,^9^. Consistent with our results, Somleva *et al.* demonstrated that the *SERK* gene was expressed in a subpopulation of cells competent to form somatic embryos during the induction of embryogenic cell formation in *D.glomerata*^57^. Hecht *et al.* confirmed the *SERK1* could enhance the ability of suspension cells to undergo embryogenesis by ectopic overexpression in *Arabidopsis*^58^.

Additionally, several genes encoding enzymes involved in embryo development have been validated by qRT-PCR in the present study, including *GST*, *GAT* and *GS1-4* with high expression at 15 DAP (Fig. 6), which were involved in controlling seed development^59^,^60^. Similarly, in triticale, Grabowska *et al.* demonstrated that during the early phase of seed development, the activity of glutamine synthetase with a high level was observed as early as 3 day after flowering (DAF) and increased and reached its maximum at 15 DAF^59^, consisting with our result. *GST*s induced by various exogenous factors such as ethylene and auxins have also been shown to act as modulators of signal transduction pathways that control cell proliferation, which were up-regulated during the early events of somatic embryogenesis in a variety of plant species^31^,^60^ and zygotic embryogenesis in our study. However, the regulation mechanism and functions of these genes during the developmental process of embryos needs to be further explored.

In conclusion, this is the first study to characterize the DEGs expression profiling during radish embryogenesis and seed development with RNA-seq technology. A total of 5,777 DEGs were detected by pairwise comparisons among three cDNA libraries, including genes involved in secondary metabolism, signal transduction, hormone response/transduction and development in plants. Several identified pathways such as plant hormone signal transduction, metabolism, embryonic axis specification, embryo development and morphogenesis were involved in radish embryogenesis. Furthermore, a model of regulatory relationship between DEGs and miRNAs was proposed that might be central to radish embryogenesis and seed development. Our findings not only provide putative components and regulatory network associated with radish embryogenesis, but also facilitate further dissection of the molecular mechanisms underlying embryo morphogenesis and seed development in root vegetable crops.

## Methods

### Plant materials

Immature ovules and zygotes (0, 4, 6, 7, 10, 12, 13 and 15 DAP) from a radish (*Raphanus sativus* L.) advanced inbred line ‘NAU-DY13’ (early bolting and flowering) were randomly collected from self-pollinated ovaries growing in a growth chamber with a 16 h light at 26 °C/8 h dark at 20 °C cycle and immediately frozen in liquid nitrogen and stored at −80 °C for further use.

### DGE library construction and Illumina sequencing

The tissue samples from zygotes of 0 day after pollination, 4–7 days after pollination and 10–15 days after pollination were separately pooled into three collections for RNA preparation. Total RNA from each collection was extracted using Trizol^®^ Reagent (Invitrogen) following the manufacturer’s instructions. Library construction was carried out at Beijing Genomics Institute (BGI, Shenzhen, China) using Illumina’s DGE tag profiling technology. In brief, 6 μg of total RNA was used for mRNA capture with magnetic oligo (dT) beads, and then the first and second strand cDNA was synthesized. After purification and adaptor ligation, PCR amplification was performed for library construction. Finally, the qualified and quantified sample libraries were sequenced via Illumina HiSeq^TM^ 2500 platform.

### Data processing and digital tag profiling

The clean tags were obtained by removing 3′ adaptor fragments, low-quality tags, and several types of impurities from the raw reads. Then, the clean tags were mapped to the radish reference sequences containing genomic survey sequences (GSS), EST sequences and our mRNA transcriptome sequences by SOAP2. No more than two mismatches were allowed in the alignment^61^. Statistical analysis was performed to identify differentially expressed genes (DEGs) among different libraries using a rigorous algorithm described previously^62^. The expression level for each gene was determined by the reads number uniquely mapped to the specific gene and the total number of uniquely mapped reads in the library. Gene expression was normalized using the Reads Per kb per Million (RPKM) method^63^.

### Differentially expressed gene analysis

Regarding the significance of digital gene expression profiles^62^, a rigorous algorithm supplied from BGI was used to compare the differences in gene expression between each two DGE libraries. The threshold *P*-values were adjusted by the multiple testing procedures described by Benjamini and Yekutieli^64^ by controlling FDR (false discovery rate). In this study, FDR ≤ 0.001 and the absolute value of |log_2_Ratio| ≥ 1 were used as the threshold for judging significance difference of the gene expression.

### Functional annotation of DEGs

The DEGs were subjected to Gene Ontology (GO) database (http://www.geneontology.org/) and mapped to the reference canonical pathways in the Kyoto Encyclopedia of Genes and Genomes (KEGG). The target sequences were allocated to the corresponding functional categories on the basis of the BLAST searches by GO annotation using default parameters. The gene expression patterns of each pairwise comparison (0_DAP-vs-7_DAP, 0_DAP-vs-15_DAP, 7_DAP-vs-15_DAP) was analyzed and genes were clustered according to their expression level using self-organizing map using Cluster 3.0 with all the default parameters except the Euclidean distance of similarity metric. Additionally, the expression values were log_2_-transformed. Heat-maps with cluster data were then constructed using Java Tree View (http://jtreeview.sourceforge.net/) for visualization of the hierarchical clustering results^65^.

### Quantitative real-time RT-PCR validation

Quantitative real-time PCR (qRT-PCR) was employed to validate the results from high-throughput sequencing of the differentially expressed genes. Total RNAs were obtained from five radish samples (0, 4, 7 and 15 DAP) as described above. All reactions were performed on an iCycler iQ real-time PCR detection system (BIO-RAD) with three biological and three technological replications, respectively, which were carried out in a total volume of 20 μl including 0.2 μM primer pairs, 2 μl diluted cDNA and 10 μl 2×SYBR Green PCR Master Mix (TaKaRa Bio Inc., Dalian, China). The amplification reactions were incubated at 95 °C for 30 s, followed by 40 cycles of 95 °C for 5 s, 58 °C for 15 s, and 72 °C for 20 s. The *Actin* gene was selected as the internal control. Relative gene expression levels were calculated using the 2^−△△*C*t^ method. The primers for the selected transcripts were shown in Supplementary Table S9.

### Correlation analysis between radish embryogenesis-related miRNAs and DEGs

The correlation analysis between mRNA and miRNA expression during embryogenesis in radish was performed using Cytoscape_v3.2.1 software^66^. The radish embryogenesis-associated miRNA data were obtained from our previous study^27^. Briefly, to identify the co-regulated differentially expressed miRNAs and mRNAs, the target genes of miRNA were compared with the normalized DEGs. The relationship between miRNA and mRNA was assessed according to the log_2_ value. For instance, the log_2_ value of miRNA and its corresponding gene were both above 0, and then they were up-up regulation. After establishing the contingency table, a visualization of nodes and edges as a two-dimensional network layout was constructed^67^.

## Additional Information

**How to cite this article**: Zhai, L. *et al.* Transcriptional identification and characterization of differentially expressed genes associated with embryogenesis in radish (*Raphanus sativus* L.). *Sci. Rep.* **6**, 21652; doi: 10.1038/srep21652 (2016).

## Supplementary Material

Supplementary Information

Supplementary Table S1

Supplementary Table S2

Supplementary Table S3

Supplementary Table S4

Supplementary Table S5

Supplementary Table S6

Supplementary Table S7

Supplementary Table S8

## Figures and Tables

**Table 1:** Summary of alignment statistics of RNA-seq in three libraries (0_DAP, 7_DAP and 15_DAP) mapped to reference genome.

	0_DAP		7_DAP		15_DAP	
	reads number	percentage	reads number	percentage	reads number	percentage
Total Reads	7,191,808	100.00%	7,235,080	100.00%	6,963,130	100.00%
Total BasePairs	352,398,592	100.00%	354,518,920	100.00%	341,193,370	100.00%
perfect match	3,874,571	71.05%	3,871,479	71.44%	3,557,884	70.03%
<=2bp mismatch	1,578,680	28.95%	1,547,650	28.56%	1,522,565	29.97%
unique match	4,307,081	78.98%	4,253,014	78.48%	4,048,952	79.70%
multi-position match	1,146,170	21.02%	1,166,115	21.52%	1,031,497	20.30%

**Table 2:** The enriched pathways of significantly differentially-regulated DEGs in three libraries (0_DAP, 7_DAP and 15_DAP) based on pairwise comparison.

Pathways	ID	0_DAP-vs-7_DAP		0_DAP-vs-15_DAP		7_DAP-vs-15_DAP	
		No. of DEG	*p*-Value	No. of DEG	*p*-Value	No. of DEG	*p*-Value
Starch and sucrose metabolism	ko00500	150	4.21E-18	155	3.17E-11	77	2.54E-11
Pentose and glucuronate interconversions	ko00040	84	2.29E-11	78	3.50E-05	42	7.81E-07
Plant hormone signal transduction	ko04075	216	7.96E-11	256	1.76E-09	118	1.34E-09
Ribosome biogenesis in eukaryotes	ko03008	72	1.09E-05	79	0.000538	8	0.999389
DNA replication	ko03030	33	0.000254	37	0.001201	2	0.997389
Brassinosteroid biosynthesis	ko00905	13	0.003174	9	0.252944	10	0.000297
Glycosphingolipid biosynthesis-globo series	ko00603	9	0.007287	8	0.070432	3	0.223588
Thiamine metabolism	ko00730	6	0.01125	3	0.434719	2	0.227954
Indole alkaloid biosynthesis	ko00901	12	0.020144	17	0.002195	10	0.000767
Zeatin biosynthesis	ko00908	18	0.037568	18	0.201559	16	0.000138
Vitamin B6 metabolism	ko00750	7	0.04039	8	0.05051	4	0.066465
Folate biosynthesis	ko00790	7	0.161538	3	0.927808	1	0.884103
Phenylpropanoid biosynthesis	ko00940	67	0.166402	82	0.23998	56	1.77E-06
Biotin metabolism	ko00780	2	0.20004	3	0.086867	2	0.059061
Phenylalanine, tyrosine and tryptophan biosynthesis	ko00400	16	0.805192	22	0.729426	15	0.046203
Glycerophospholipid metabolism	ko00564	25	0.833352	35	0.70619	9	0.945405
Glycine, serine and threonine metabolism	ko00260	24	0.90091	34	0.802886	26	0.003606
Alanine, aspartate and glutamate metabolism	ko00250	22	0.940543	33	0.807366	24	0.009459
Fatty acid biosynthesis	ko00061	8	0.96842	35	0.000121	−	−
Glutathione metabolism	ko00480	25	0.997484	38	0.985319	12	0.975054

**Table 3:** The identified genes involved in plant hormone signal transduction pathway.

Entry	Gene Name	Definition	E. C. Number
K13946	*AUX1*	auxin influx carrier (AUX1 LAX family)	
K14485	*TIR1*	transport inhibitor response 1	
K14484	*AUX/IAA*	auxin-responsive protein IAA	
K14486	*ARF*	auxin response factor	
K14487	*GH3*	auxin responsive GH3 gene family	
K14488	*SAUR*	SAUR family protein	
K14489	*CRE1*	Arabidopsis histidine kinase 2/3/4 (cytokinin receptor)	EC:2.7.13.3
K14490	*AHP*	histidine-containing phosphotransfer peotein	
K14491	*B-ARR*	two-component response regulator ARR-B family	
K14492	*A-ARR*	two-component response regulator ARR-A family	
K1449	*GID1*	gibberellin receptor GID1	EC:3.-.-.-
K14495	*GID2*	F-box protein GID2	
K14494	*DELLA*	DELLA protein	
K1451	*CTR1*	serine/threonine-protein kinase CTR1	EC:2.7.11.1
K14512	*MPK6*	mitogen-activated protein kinase 6	EC:2.7.11.24
K14513	*EIN2*	ethylene-insensitive protein 2	
K14515	*EBF1/2*	EIN3-binding F-box protein	
K14516	*ERF1/2*	ethylene-responsive transcription factor 1	
K13416	*BAK1*	brassinosteroid insensitive 1-associated receptor kinase 1	EC:2.7.10.12.7.11.1
K13415	*BRI1*	protein brassinosteroid insensitive 1	EC:2.7.10.1 2.7.11.
K1450	*BSK*	BR-signaling kinase	EC:2.7.11.
K14503	*BZR1/2*	brassinosteroid resistant 1/2	
K14504	*TCH4*	xyloglucan:xyloglucosyl transferase TCH4	EC:2.4.1.207
K14505	*CYCD3*	cyclin D3, plant	
