# Supplementary material for: Transcriptional identification and characterization of differentially expressed genes associated with embryogenesis in radish (Raphanus sativus L.)
Source: Sci Rep. 2016 Feb 23;6:21652. doi: 10.1038/srep21652 (PMC4763228; doi:10.1038/srep21652)
Supplement: Supplementary Information [file srep21652-s1.pdf]

Supplementary information

Transcriptional identification and characterization of differentially expressed genes associated with embryogenesis in radish (*Raphanus sativus* L.)

Lulu Zhai[1,2], Liang Xu[1], Yan Wang[1], Xianwen Zhu[3], Haiyang Feng[1], Chao Li[1], Xiaobo Luo[1], Muleke Everlyne[1], Liwang Liu[1,*]

1. Supplementary Figures

Supplementary Figure S1: Distribution coverage of DEGs. A: Library 0_DAP, B: Library 7_DAP and C: Library 15_DAP.

Supplementary Figure S2: Comparison of gene expression levels among the three libraries based on pairwise comparison. A, B and C showed gene expression level of 0_DAP vs 7_DAP, 0_DAP vs 15_DAP and 7_DAP vs 15_DAP, respectively. With an estimated false-discovery rate (FDR) of$\leq$0.001 and$|log_2Ratio|\geq1$, the red shows the proportion of up-regulated genes; the green represents down-regulated genes, and the blue shows the proportion of genes not differentially expressed.

Supplementary Figure S3: The related genes involved in pathway of plant hormone signal transduction. The pathway originate from Kyoto Encyclopedia of Genes and Genome data base; A: DEGs between 0_DAP and 7_DAP library; B: DEGs between 0_DAP and 15_DAP library; C: DEGs between 7_DAP and 15_DAP library.

Supplementary Fig. S4: Comparison of expression profile by RNA-Seq and RT-qPCR. Comparison of expression profiles of 17 randomly selected genes by RNA-seq and RT-qPCR showing different expression profiles in three comparative groups (0_DAP_vs_7_DAP, 0_DAP_vs_15_DAP and 7_DAP_vs_15_DAP) during embryogenesis. Each data point represent the log2 normalized expression level obtained from RNA-seq (y axis) and RT-qPCR (x axis) analyses.

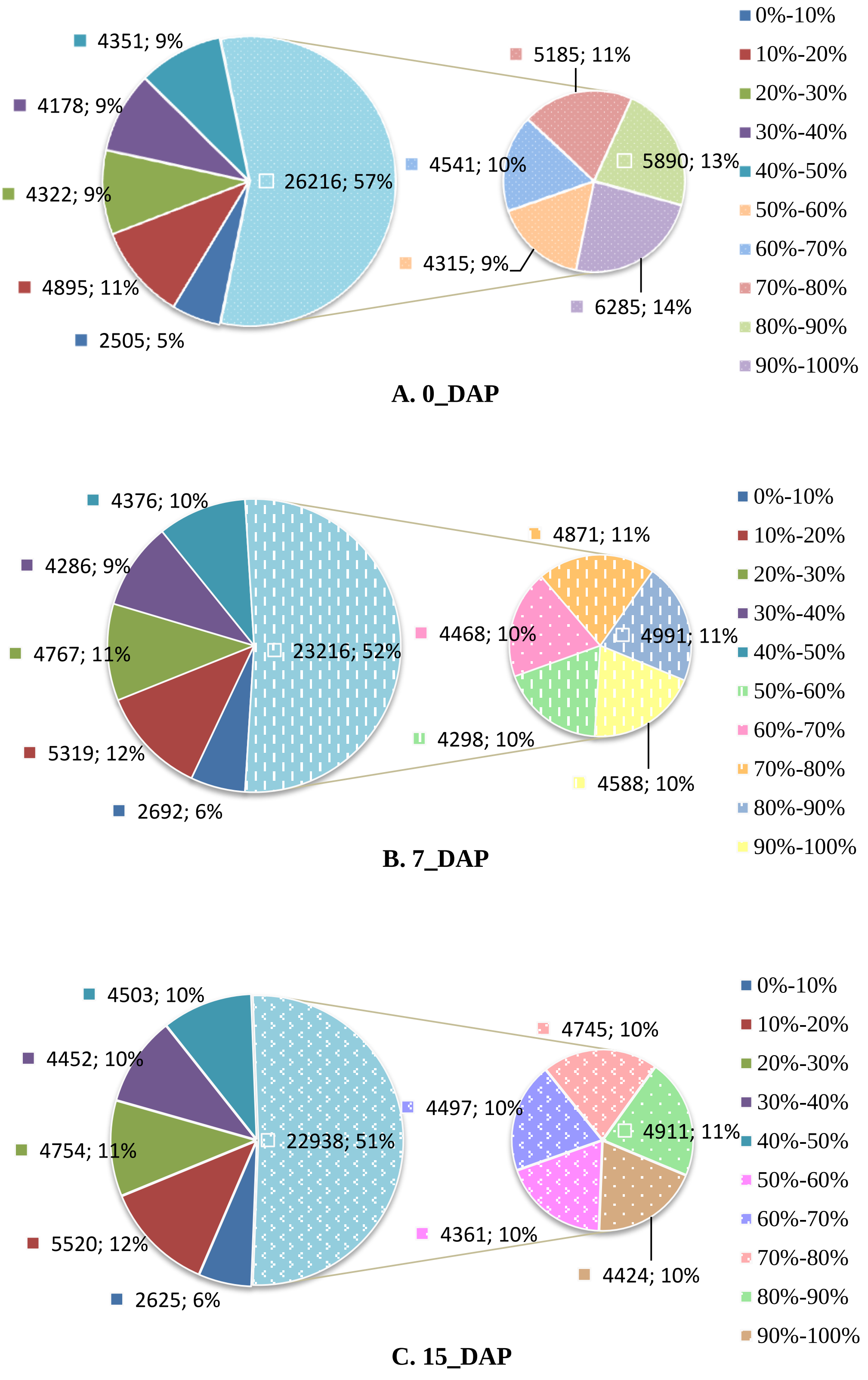

Supplementary Figure S1: Distribution coverage of DEGs. A: Library 0_DAP, B: Library 7_DAP and C: Library 15_DAP.

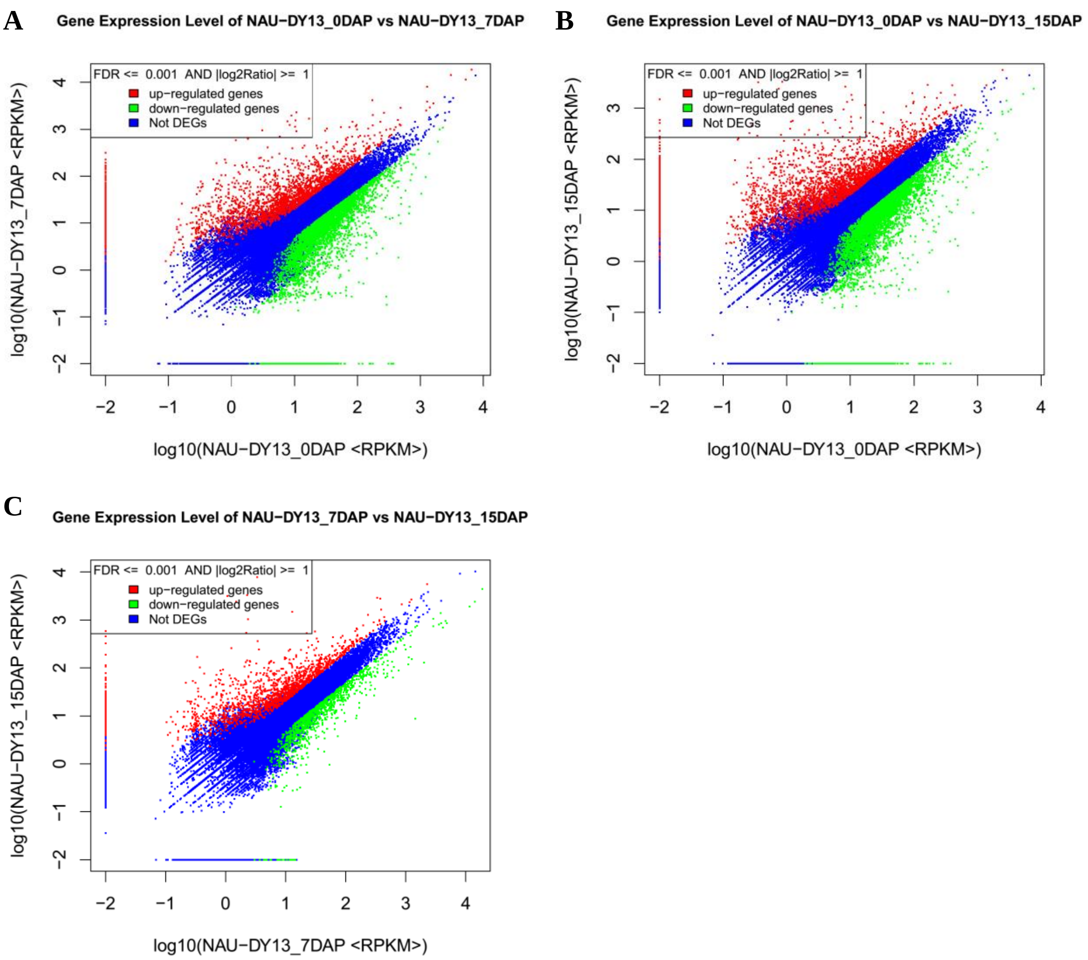

Supplementary Figure S2: Comparison of gene expression levels among the three libraries based on pairwise comparison. A, B and C showed gene expression level of 0_DAP vs 7_DAP, 0_DAP vs 15_DAP and 7_DAP vs 15_DAP, respectively. With an estimated false-discovery rate (FDR) of≤0.001 and|log_2Ratio|≥1, the red shows the proportion of up-regulated genes; the green represents down-regulated genes, and the blue shows the proportion of genes not differentially expressed.

A

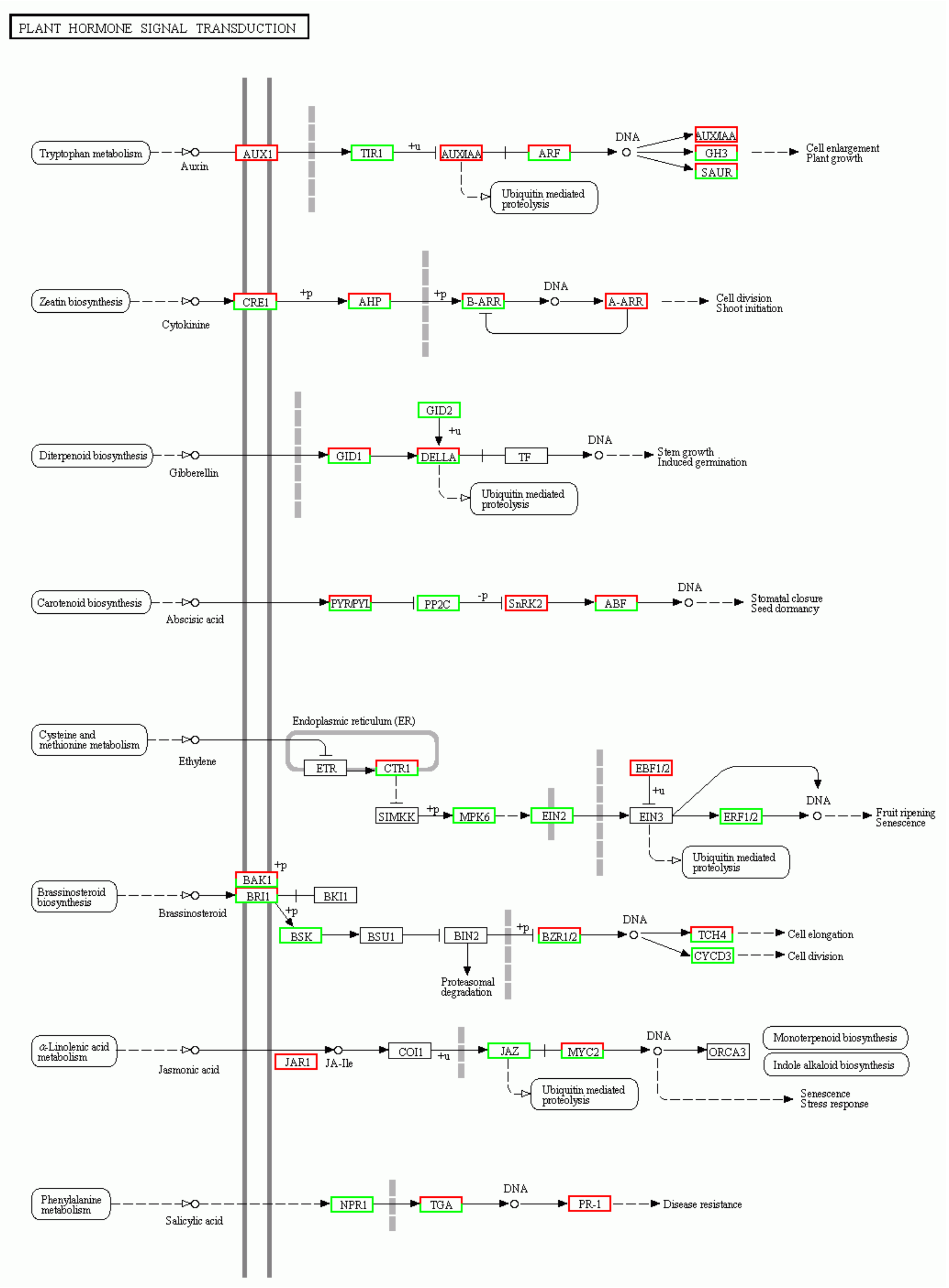
PLANT HORMONE SIGNAL TRANSDUCTION
Tryptophan metabolism
Auxin
AUX1
TIR1
+u
AUX/IAA
ARF
DNA
AUX/IAA
GH3
SAUR
Cell enlargement
Plant growth
Ubiquitin mediated proteolysis
Zeatin biosynthesis
Cytokinine
CRE1
+p
AHP
+p
B-ARR
DNA
A-ARR
Cell division
Shoot initiation
GID2
+u
Diterpenoid biosynthesis
Gibberellin
GID1
DELLA
TF
DNA
Stem growth
Induced germination
Ubiquitin mediated proteolysis
Carotenoid biosynthesis
Abscisic acid
PYR/PYL
PP2C
-p
SnRK2
ABF
DNA
Stomatal closure
Seed dormancy
Endoplasmic reticulum (ER)
Cysteine and methionine metabolism
Ethylene
ETR
CTR1
SIMKK
+p
MPK6
EIN2
EBF1/2
+u
EIN3
ERF1/2
DNA
Fruit ripening
Senescence
Ubiquitin mediated proteolysis
+p
BAK1
BRI1
BKI1
Brassinosteroid biosynthesis
Brassinosteroid
+p
BSK
BSU1
BIN2
+p
BZR1/2
DNA
TCH4
CYCD3
Cell elongation
Cell division
Proteasomal degradation
α-Linolenic acid metabolism
Jasmonic acid
JAR1
JA-Ile
COI1
+u
JAZ
MYC2
DNA
ORCA3
Monoterpenoid biosynthesis
Indole alkaloid biosynthesis
Ubiquitin mediated proteolysis
Senescence
Stress response
Phenylalanine metabolism
Salicylic acid
NPR1
TGA
DNA
PR-1
Disease resistance

B

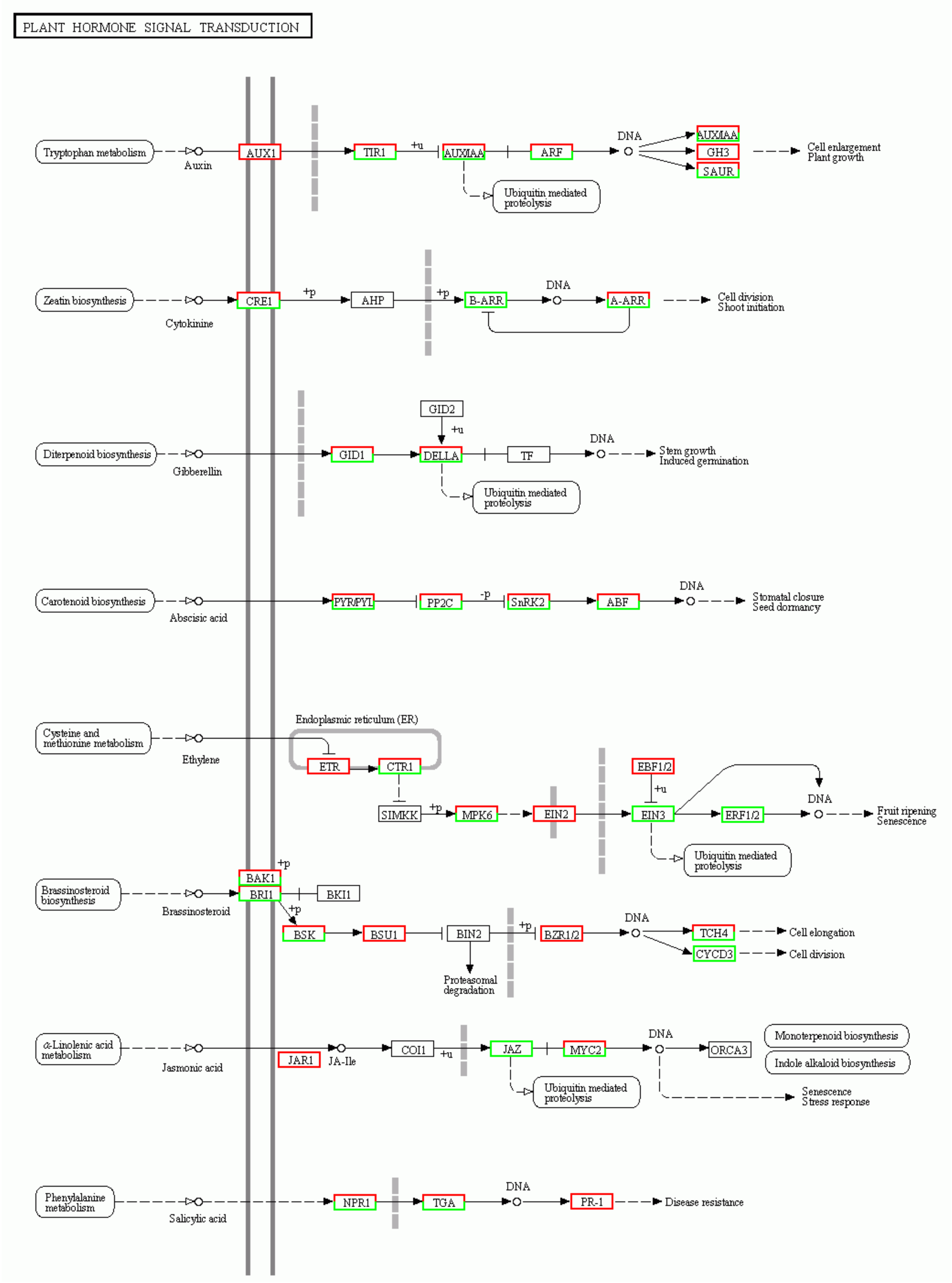
PLANT HORMONE SIGNAL TRANSDUCTION
Tryptophan metabolism
Auxin
AUX1
TIR1
+u
AUX/IAA
ARF
DNA
AUX/IAA
GH3
SAUR
Cell enlargement
Plant growth
Ubiquitin mediated proteolysis
Zeatin biosynthesis
Cytokinine
CRE1
+p
AHP
B-ARR
A-ARR
Cell division
Shoot initiation
GID2
Diterpenoid biosynthesis
Gibberellin
GID1
DELLA
TF
Stem growth
Induced germination
Carotenoid biosynthesis
Abscisic acid
PYR/PYL
PP2C
-p
SnRK2
ABF
Stomatal closure
Seed dormancy
Endoplasmic reticulum (ER)
Cysteine and methionine metabolism
Ethylene
ETR
CTR1
SIMKK
MPK6
EIN2
EBF1/2
EIN3
ERF1/2
Fruit ripening
Senescence
Brassinosteroid biosynthesis
Brassinosteroid
BAK1
BRI1
BKI1
BSK
BSU1
BIN2
BZR1/2
Proteasomal degradation
TCH4
CYCD3
Cell elongation
Cell division
α-Linolenic acid metabolism
Jasmonic acid
JAR1
JA-Ile
COI1
JAZ
MYC2
ORCA3
Monoterpenoid biosynthesis
Indole alkaloid biosynthesis
Senescence
Stress response
Phenylalanine metabolism
Salicylic acid
NPR1
TGA
PR-1
Disease resistance

C

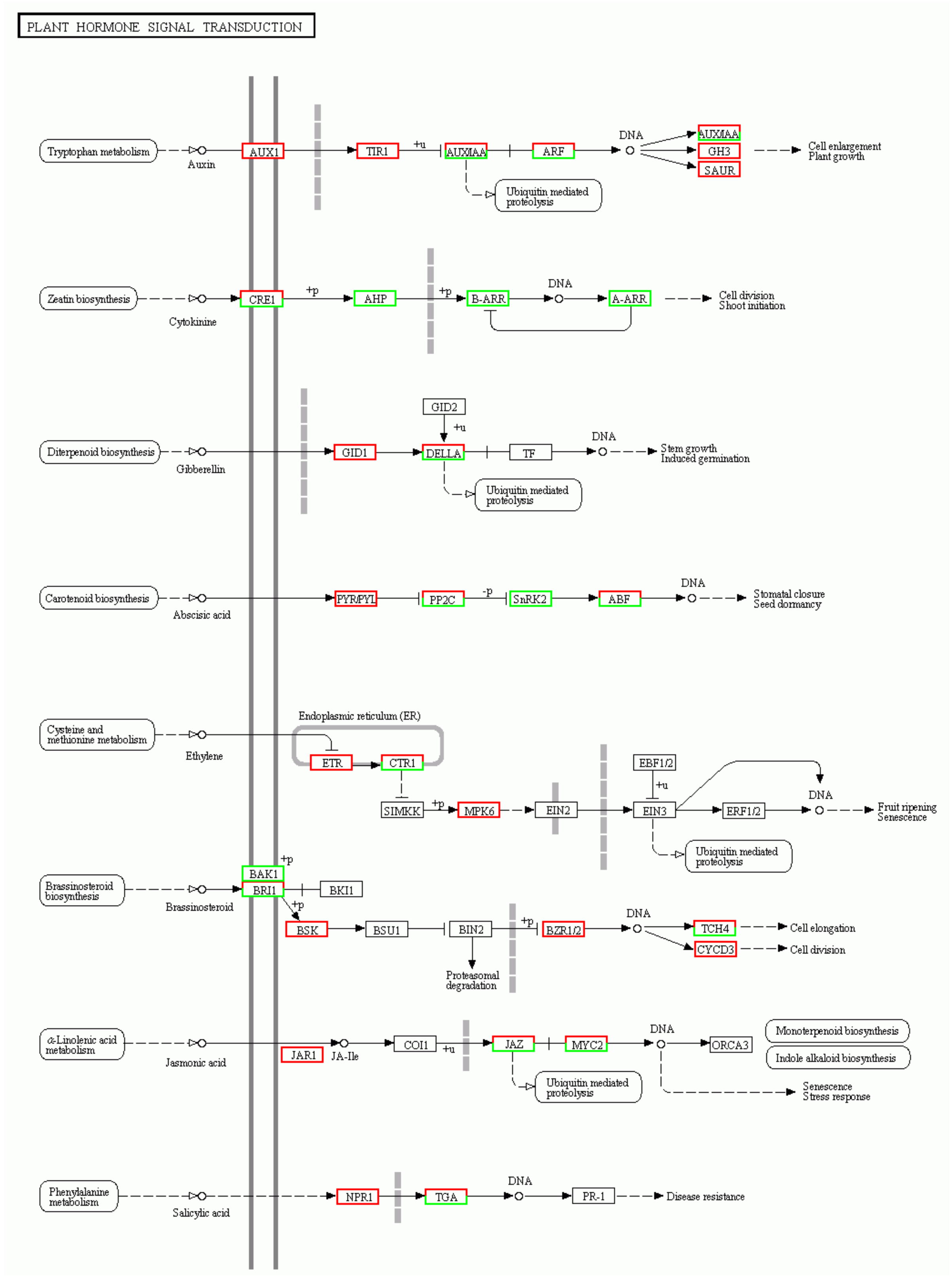

Supplementary Figure S3: The related genes involved in pathway of plant hormone signal transduction. The pathway originate from Kyoto Encyclopedia of Genes and Genome data base; A: DEGs between 0_DAP and 7_DAP library; B: DEGs between 0_DAP and 15_DAP library; C: DEGs between 7_DAP and 15_DAP library.

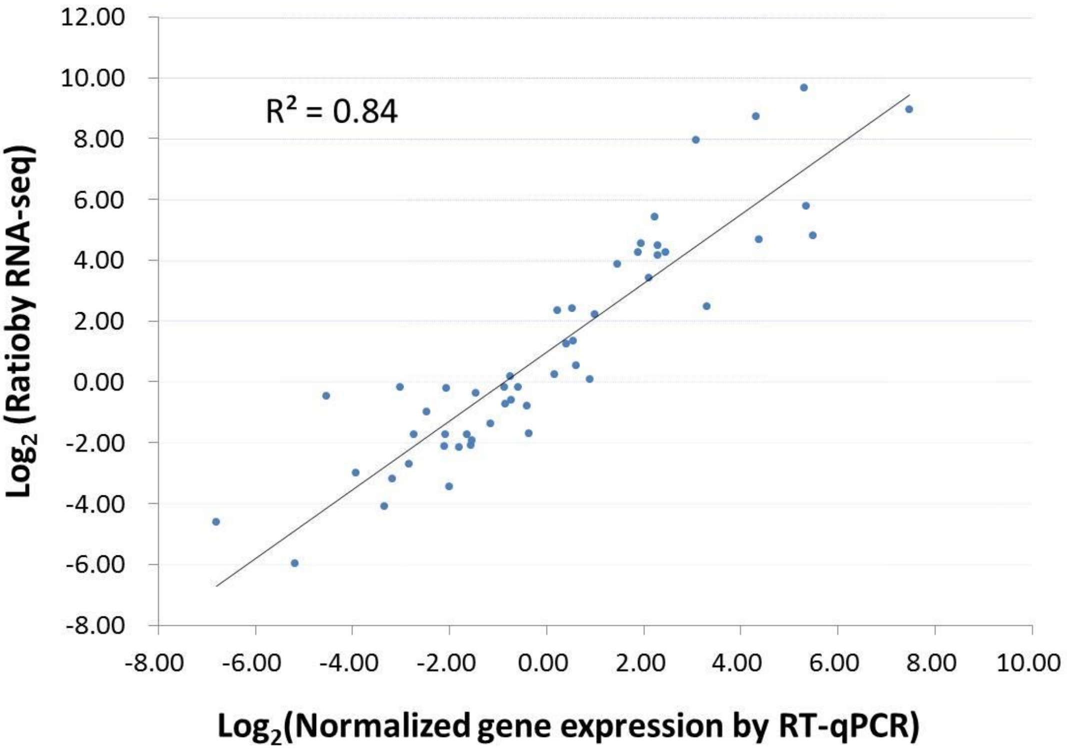

Supplementary Fig. S4: Comparison of expression profile by RNA-Seq and RT-qPCR. Comparison of expression profiles of 17 randomly selected genes by RNA-seq and RT-qPCR showing different expression profiles in three comparative groups (0_DAP_vs_7_DAP, 0_DAP_vs_15_DAP and 7_DAP_vs_15_DAP) during embryogenesis. Each data point represent the $\log_2$ normalized expression level obtained from RNA-seq (y axis) and RT-qPCR (x axis) analyses.

2. Supplementary Tables

Supplementary Table S1: The expressed genes with coverage more than 50% (See Excel file).

Supplementary Table S2: The up- and down-regulated transcripts based on pairwise comparison among DGE libraries (See Excel file).

Supplementary Table S3: The list of DEGs among three cDNA libraries (See Excel file).

Supplementary Table S4: GO functional enrichment analysis at early stage of embryogenesis (See Excel file).

Supplementary Table S5: KEGG pathways analysis based on pairwise comparison (See Excel file).

Supplementary Table S6: The DEGs involved in embryo induction, formation and maturation stages (See Excel file).

Supplementary Table S7: Expression profiles of certain interested genes involved during radish embryogenesis (See Excel file).

Supplementary Table S8: The correlation analysis between DEGs and miRNAs during radish embryogenesis (See Excel file).

Supplementary Table S9: The primers of differentially-regulated genes for RT-qPCR analysis.

Supplementary Table S9: The primers of differentially-regulated genes for RT-qPCR analysis.

Unigene	Gene name	Abbreviation name	Primer sequence		Length (bp)
			Forward sequence (5'-3')	Reverse sequence (5'-3')	
CL11244.Contig1_CKA	WRKY transcription factor 22	WRKY22	TTTCTCTCTCTGCTTCTTC	TTCTGGTGGTTACTGTTG	123
CL12457.Contig1_CKA	efflux carrier of polar auxin transport	ECPAT	AATGGAGATAGATTGCTGAA	CTGAGAATGGACTGAACA	263
CL14709.Contig1_CKA	Fus3-complementing gene 1	FC1	ATTTCCTTCCTCTCCCTAT	CTGTTGCTCGTAATGATTC	166
CL203.Contig3_CKA	auxin response factor 3-1	ARF3-1	TGGCTCATCATCATCATC	TTCATCAACACCTCTTCG	182
CL5790.Contig1_CKA	WRKY transcription factor 2	WRKY2	ACCAACCATTACCAAGAG	AAACGCTAAACCAGGAAT	174
CL6021.Contig2_CKA	late embryogenesis abundant	LEAHRG	CTTGTCGTAGTGAATGGA	CATCGCTTTAATACTCTATCTC	172
CL6154.Contig1_CKA	Argonaute/Zwille-like protein 2	AG/ZLL2	TCTTCTCCTTCTCCATCA	TTTCTCTCTTCCCGTTTC	184
CL6810.Contig1_CKA	aintegumenta	AIL	CGGAAGCCTATGATGTAG	TCGTTGTTGTTGTTGTTG	172
CL7947.Contig4_CKA	Suppressor of ABI3-5	SUA	GCTCCTCCTATTGACATC	TTATCCTCGTGAACTTCG	283
CL878.Contig7_CKA	ferritin	FERRITIN	GACATAACAGACCGAGAG	CATCATCATCAACAATCACTA	186
CL9728.Contig1_CKA	Class I glutamine amidotransferase	GAT	TGGTGGTTGCTAATGATAA	GAGAGTCCTTGTTGTTGT	292
gi|166138539|gb|FD570215.1	AGL9	AGL9	TGAAGATGATAGAGAACAAGAT	GAGAAGATGATGAGAGCAA	124
gi|166144826|gb|FD576744.1	F-box/LRR-repeat protein	FBXL	TCTCAACTTCTCCGATTATC	ACACCTTACCAACATCATT	200
gi|167442117|gb|FD948074.1|FD948074	auxin efflux carrier family protein	EIR	CAGTTGGTGCGATTATTG	CGATGATTGGGAGAAGTAT	243
gi|167451596|gb|FD956186.1|FD956186	baby boom interacting protein 2	BIP2	GTTCTTCTTCAGGCTGTA	TTCATCGTATTCCGTGTG	104
gi|332783910|gb|FY434046.1	ARF8-1	ARF8-1	TGGACAAGGAGGATGATA	AGATAACACAAGGGAAACA	212
gnl|UG|Rsa#S42001404	Small auxin up RNA protein	SAUR	GAACGGACATCATCTCTC	AATCAGTAGCAACGACAA	270
gnl|UG|Rsa#S42026394	Late embryogenesis abundant	LEA	CTAACCGCTATGCCATAA	GAATCCTTACTCTCAATCCA	165
gnl|UG|Rsa#S42049349	Late embryogenesis abundant protein	LEA	TAAGCACAAGGATGAACA	AACCACTATGAACCAGAAA	276
gnl|UG|Rsa#S43005757	Auxin responsive like protein	ARL	GTGGTAAGAGAAGGTAAGG	ATCAGTTTGGGAGCATTT	170

Supplementary Table S9: **The primers of differentially-regulated genes for RT-qPCR analysis. (Continue)**

Unigene	Gene name	Abbreviation name	Primer sequence		Length (bp)
			Forward sequence (5’-3’)	Reverse sequence (5’-3’)	
gnl|UG|Rsa#S43013377	Glutathione S-transferase	GST	TACTTGGTGGGATGAAAC	CCTGACGAACTCTTAACA	299
Unigene13496_CKA	SAUR-like auxin-responsive protein	SAUR	GATGATGATGATGAAGAAGATT	TTCCATATAAACCTCCACAA	170
Unigene18591_CKA	Zwille-like protein 2	ZLL2	ATCCCTCAAACAACCATT	ATCATAACAGCGAACTCTT	185
Unigene1912_CKA	glutamate decarboxylase 1	GAD1	GTCGGATAAGTTGGTAGTAT	AAGTGGAGGATTCATAGC	266
Unigene21980_CKA	Probable LRR receptor-like	LRR-STK	GATACTGTAGGCTCTGAAG	GTTCCTTGACCTTTCCTC	149
Unigene23299_CKA	Argonaute-like protein	AGL	CGAGACACAGTTCAAGAA	ATGCGTTATCACCGTATC	196
Unigene25317_CKA	Leucine-rich repeat-containing	LRRC	ATCTTCATCCACTACCTCT	CCAGCGTTCTTCTTCTTA	164
Unigene25469_CKA	Glutamine synthetase cytosolic isozyme 1-4	GS1-4	CAGGCTTGGATATGAGAA	GCTTGAGGGTATATGATGA	142
Unigene27378_CKA	Nuclear factor Y	NF-Y	AACGGAAGAAGAAGAAGAA	TGGTGAGGTGTAGAAGAT	167
Unigene28056_CKA	CHD3-type chromatin-remodeling	PKL	AGCACAGAATAACCAGAAT	CCATAATAATCCTCCGCATA	192
Unigene31774_CKA	embryonic flower 2_2	EMF2_2	GCATCTCTTCCTCTTCAC	ATCTTCAGTTACTTCAGTCTT	110
Unigene4179_CKA	Leafy cotyledon 1	LEC1-2	CAGGTAGTGGTCTTGTTC	TCTTGATACGCTTTCTTAATAC	195
Unigene446_CKA	Embryo sac development arrest 7	EDA7	CTTCCTCTGACAACATCC	CTTTCCACAACGGTAGAT	262
